# Technical and Anatomical Aspects of Retroperitoneoscopic Renal Surgery: A Summary of Tribulations and Resolutions Encountered at a Tertiary Care Institute of North India

**DOI:** 10.7759/cureus.59380

**Published:** 2024-04-30

**Authors:** Amit Mishra, Amit Kumar Shreevastava, Rajat S Das

**Affiliations:** 1 Urology, All India Institute of Medical Sciences, Raebareli, IND; 2 Anatomy, All India Institute of Medical Sciences, Raebareli, IND

**Keywords:** nephrectomy, renal, retroperitoneoscopy, laparoscopy, kidney

## Abstract

Introduction: Kidneys are a retroperitoneal organ but the widely practiced laparoscopic approach to renal surgery is transperitoneal due to the advantages of greater working space at the cost of entering the peritoneal cavity, risk of injury to intraperitoneal organs, and the increased risk of postoperative bowel complications. The classic open approach to kidney procedures has been the flank approach without violating the peritoneal cavity instead of the retroperitoneal approach to renal surgery with the advantages of direct access to the renal hilum, especially the renal artery. Being a technically challenging procedure, the retroperitoneoscopic approach is less practiced and needs an experienced surgical team. Through this study, we have tried to unveil the myths and illustrate the exact position of ports, which is the decisive initial step in retroperitoneoscopic surgery.

Material and methods: This retrospective study was conducted at a developing tertiary center in northern India with novice staff mainly to determine the technical and anatomical caveats pertaining to the retroperitoneoscopic approach for renal surgeries, the challenges faced, and their resolutions. The decision for the site of incision for primary or camera port was taken only after a proper anatomical study of the cadavers and ongoing retroperitoneal surgical experience while treating various patients suffering from renal diseases.

The study comprised eight patients, during the period from June 2023 to March 2024. Various parameters, such as demographic variables, diagnosis, mean operative time, estimated blood loss, technical difficulties encountered and their resolution, complications, and reasons for conversion were studied. A total of 15 cadavers were dissected during the above time period to study finer anatomical details of port positioning and other details.

Results: After an elaborate study of 15 cadavers and thereafter performing surgery on eight patients during the above time period, surgery was successfully performed on six patients, and two patients needed conversion to open procedure due to dense adhesions and non-progression while complications occurred in two patients (peritoneal rent and renal vein injury), which were managed laparoscopically.

Conclusion: Nonetheless, restrictions of surgical space make retroperitoneoscopic space a challenging procedure but with elaborate experience, which we gained through cadaveric study, and surgical results obtained during the initial few cases such as the exact site of the primary port and technical intricacies, and handling of complications if and when faced, we hope our study will certainly make retroperitoneal space more amicable to urologists.

## Introduction

There are two basic techniques of laparoscopic nephrectomy. One is the transperitoneal approach, which is practiced worldwide, and the other is the retroperitoneoscopic approach, which is less commonly practiced. The latter has more advantages, is safer, and provides easy access to renal vasculature but it is not performed routinely owing to its complexity and arduous learning curve caused by a lack of clear-cut guiding principles on the technique [1]. In 1969, Bartel first described the technique of retroperitoneoscopy, which at that time was considered to be technically challenging and thus not feasible due to restricted working space, absence of well-defined anatomical landmarks, and abundant adipose tissue in the retroperitoneum [2]. The landmark discovery of atraumatic balloon dissection of the retroperitoneum by Dr. Gaur made this procedure more expedient [3].

Novelty: Unveiling the myth

To date, all previous researchers have mentioned the 12th rib as a landmark, which is not a constant structure and leads to faulty positioning of the primary camera port and massive bleeding from advancement into the psoas muscle if the 12th rib is too short [4]. At the same time, an overly anterior position of the primary port leads to much intraoperative difficulty. For safer and more successful execution of the surgery, we have highlighted the minute technical and anatomical details of positioning and direction of insertion of the balloon dilator, positioning of anterior port posterior to the anterior axillary line, and positioning and direction of insertion of the renal angle port, troubleshooting the technical difficulties encountered during the procedure.

## Materials and methods

The main objective behind this study was to emphasize the technical and anatomical caveats pertaining to the retroperitoneoscopic approach for renal surgeries and not the results of the procedure that have already been extensively studied. Identification of anatomical planes and the technique for creating the primary port (camera port), which is the most important step, have been vaguely described in medical literature. The decision on the site of incision for the primary or camera port was taken only after a proper anatomical study of the cadavers and ongoing retroperitoneal surgical experience while treating various patients suffering from renal disease. Using this study, we would also like to highlight the trials and tribulations faced and our resolution thereof with regard to this seldom performed procedure, especially in a developing tertiary healthcare center with amateur staff having no previous exposure to this procedure.

The study included eight patients during the period of June 2023 to February 2024. All patients were scheduled for surgery after the nonfunctioning status of the kidney was confirmed with a renal scan and proper informed consent for surgery was obtained. Patients with a history of previous open renal surgery, renal abscess, and percutaneous nephrostomy tube were excluded from the study. The data were analyzed in a retrospective and deidentified manner from patient case records. We studied the following parameters: demographic variables, diagnosis, mean operative time, estimated blood loss, technical difficulties encountered along with their resolution, complications, and reasons for conversion.

Further, to support our study anatomically, in collaboration with the Department of Anatomy, 15 cadavers were dissected during the aforementioned time period, and the exact site for the primary or camera port and layers encountered were confirmed.

Technical details

Position

The patient is placed in the extended flank position, the kidney bridge is elevated, and the operating table is flexed to maximize the space between the costal margin and the iliac crest. This limited area comprises the access “portal” for retroperitoneoscopic surgery [5]. The shoulder and iliac crest should be in the same line and properly strapped; otherwise, the patient's upper torso will either fall forward or backward, leading to a faulty port position. The contralateral leg should be flexed at the knee joint, and the ipsilateral leg should be extended to keep the tensor fascia lata taut to ensure sufficient space. The materials used include a low-cost balloon, which can be made from the middle finger of gloves (doubly ligated with silk 1-0 over a K-90 catheter), a pediatric Langenbeck retractor, a 50 ml syringe for saline infusion, and routine laparoscopy instruments.

Incision for the Primary Port

**Figure 1:**
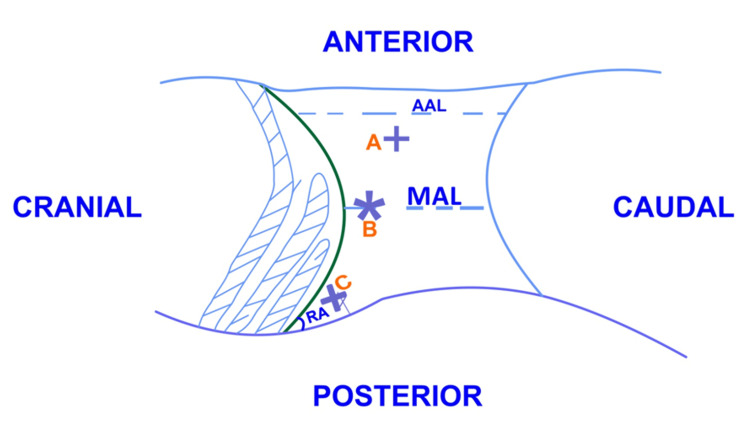
Anatomical landmarks for a primary incision for camera port (B) and accessory ports (A & C). Schematic representation of surface markings in the right lateral decubitus position prior to surgery. AAL: anterior axillary line; MAL: mid-axillary line; RA: renal angle. Figure created by the authors.

The initial access is obtained by the open technique. A horizontal 1.5 to 2-cm skin incision is created 1 cm below the point where the costal margin intersects with the mid-axillary line at the highest point in the flank position (Figure 1). Next, the flank muscle fibers are incised in layers with the help of electrocautery and bluntly separated with pediatric Langenbeck retractors. Entry is gained into the retroperitoneum by gently piercing the anterior thoracolumbar fascia with the scissor or cautery, allowing us to see the glistening pararenal fat moving with respiration. This is the endpoint.

Creation of Pneumoretroperitoneum

**Figure 2:**
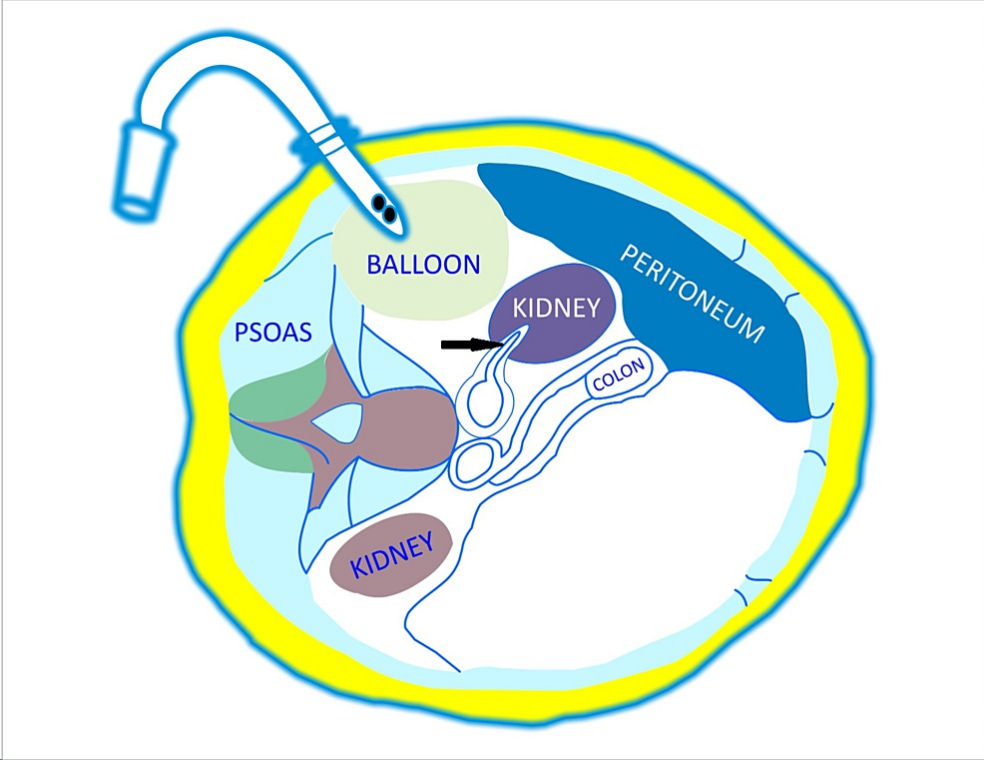
Dr. Gaur's balloon dissection technique of the retroperitoneum. Schematic cross-sectional representation of body at L2 vertebral level. The inflated balloon has displaced the right kidney anteromedially with its vascular pedicle pushed ventrally (black arrow). Figure created by the authors.

Using a peanut sponge, the dissection is performed in the cephalad and posterior direction, remaining immediately anterior to the psoas muscle and posterior to the Gerota’s fascia (containing paranephric fat) to create a space for placement of the balloon dilator. The aim is to push the Gerota’s fascia and its contents anteriorly because if the balloon is inflated anterior to the Gerota's fascia, it will push the kidney down, leading to great intraoperative difficulty and erroneous opening of the peritoneum. Saline is inserted in the balloon according to the weight of the patient (10-12 ml/kg is adequate), creating a good working space superolaterally in the retroperitoneum (Figure 2). Following balloon deflation and removal, a 12-mm blunt-tip trocar is placed as the primary port. The pneumoretroperitoneum is created with up to 15 mmHg pressure with carbon dioxide, which rotates the right or left kidney anticlockwise or clockwise, respectively, along its longitudinal axis.

Secondary Ports

**Figure 3:**
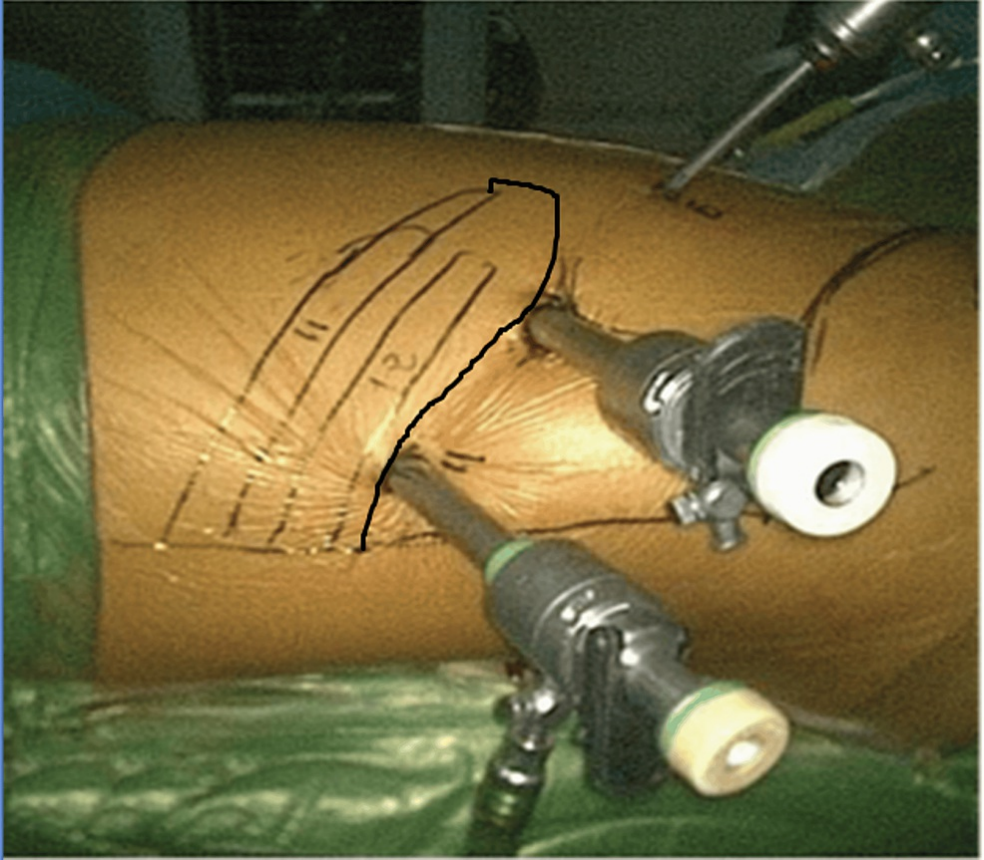
Final ports in position.

Two more ports are needed, one anterior port and the other renal angle port. For the insertion of the anterior port, the camera should be pointing 30° upward, and then under vision, a 5 mm port is placed just posterior to the anterior axillary line and about three fingers anterior to the camera port. It is important to ensure that the peritoneum has been reflected and the bare muscle is visible so that injury to the intraperitoneal structures can be prevented. For the renal angle port, a 10 mm port is placed about three fingers posterior to the camera port and lateral to the erector spinae in the renal angle. Before positioning this port, it is crucial to clear the area of fat with blunt and sharp dissection under vision, to prevent injury to adjacent structures that may also involve pleura (Figure 3). The direction of insertion of the renal angle port should be under the vision and controlled and should be directed cephalad to prevent the entry of trocar into the great vessels.

**Figure 4:**
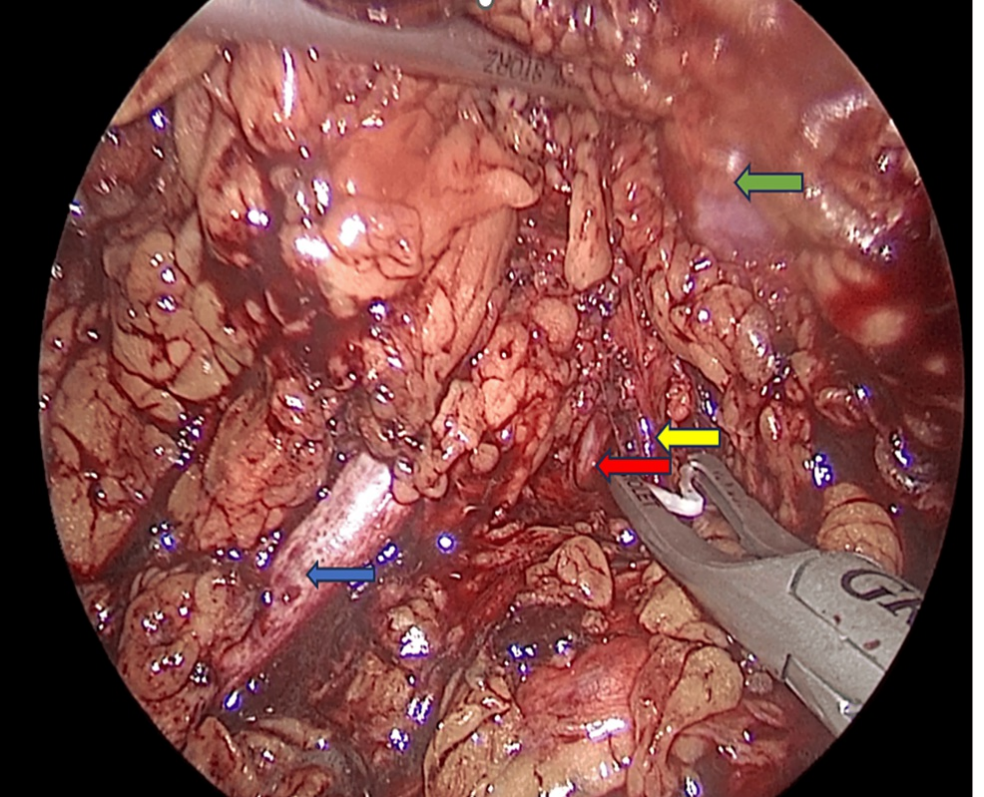
Laparoscopic view of hilar dissection. Renal artery: yellow arrow; renal vein: orange arrow; ureter: blue arrow; kidney: green arrow.

The boundary of the pneumoretroperitoneum can be described as bounded anteriorly by the fascia of Zuckerkandl and hilum, laterally by the reflected part of the peritoneum, and posteriorly by the psoas muscle and its fascia. The roof is formed by the thoracolumbar fascia along with three muscles of the abdominal wall. After port insertion, standard surgical steps follow in sequence: ureteral identification, dissection and division of the hilar vessels, and kidney mobilization (Figure 4).

Landmarks

The psoas muscle is the constant landmark. On the left side are renal artery pulsations, aorta, ureter, and gonadal vein. On the right side are renal artery pulsations, inferior vena cava (bluish-colored), and ureter.

Anatomical details

The retroperitoneum is contained anteriorly by the posterior reflection of the peritoneum and posteriorly by the abdominal wall. It is limited cranially and caudally by the diaphragm and the extraperitoneal pelvic structures, respectively. The lumbodorsal fascia merges anterolaterally with the aponeurotic part of the transversus abdominis muscle and is composed of three layers (anterior, middle, and posterior) that cover the posterior abdominal wall musculature.

During retroperitoneoscopic renal surgery, the patient should be kept in a lateral position for a better understanding of the anatomical topography of the kidney and its adjacent structures. While the patient is in the lateral position, the colon and other related viscera will descend slightly on their own due to gravity, and further during the pneumoretroperitoneum creation, the kidney is pushed more anteromedially, resulting in its forward excursion and leading to the least visceral hindrance in approaching the renal vasculature. The exact site of the incision is not fixed and may vary from surgeon to surgeon, leading to massive bleeding from muscular injury. In the present study, the exact incision site was determined by the point where the imaginary vertical midaxillary line cuts the imaginary transverse line, which passes across the lower border of the costal margin. One cm below this point, we made a transverse incision of 2-3 cm toward the anterior abdominal wall.

To reach the defined territory of the kidney, the structures encountered from superficial to deep are skin, superficial fascia, external oblique muscle, internal oblique muscle, transverse abdominis muscle, thoracolumbar fascia, paranephric fat, and renal fascia of Zuckerkandl. In our study, the incision site we chose is quite different from what was described in previous articles. Through our newer laparoscopic approach, we have minimized the complications and maximized the positive result.

## Results

Surgical results

Following our technique, a retroperitoneal space was created successfully in all cases except in one case where the balloon ruptured during saline instillation and we had to repeat the procedure. There was no significant bleeding in the working space after balloon dilatation. Out of eight cases who underwent retroperitoneoscopic nephrectomy, there were two male and six female patients with a mean age of 35 years. Indications of nephrectomy were benign renal diseases, wherein the etiology of the nonfunctioning kidney was calculus disease in four patients (two renal pelvic calculi and two ureteric calculi), pelviureteric junction obstruction in three patients, and reflux nephropathy in one patient. The mean operative duration was three hours. Blood loss was 168 ml on average. None of the patients required a blood transfusion. The complications encountered were a peritoneal breach in one case and renal vein injury in another case. In two cases, the procedure had to be converted from laparoscopic to open due to technical difficulties such as pyonephrotic changes, adhesions to the peritoneum, and nonprogression of the procedure. Duration of surgery, complication, and conversion rate were mainly dependent upon the pathological nature of the disease. The average postoperative hospital stay was six days (Table 1).

**Table 1:** Demographic variables of the patients. F: female; M: male; PUJ: pelviureteric junction.

S. No.	Age (years)/sex	Diagnosis	Operative time (in hours)	Estimated blood loss (in ml)	Hospital stay (days)
1	16/F	PUJ obstruction	3:15	200	7
2	55/F	PUJ Obstruction	2:45	200	6
3	33/F	Renal pelvic calculus	5:00	250	7
4	33/F	Reflux nephropathy	1:45	50	2
5	40/F	PUJ obstruction	3:30	150	6
6	34/M	Ureteric calculi	3:00	100	7
7	55/F	Ureteric calculi	3:00	200	7
8	21/M	Renal calculi	3:00	100	6

Anatomical results

**Figure 5:**
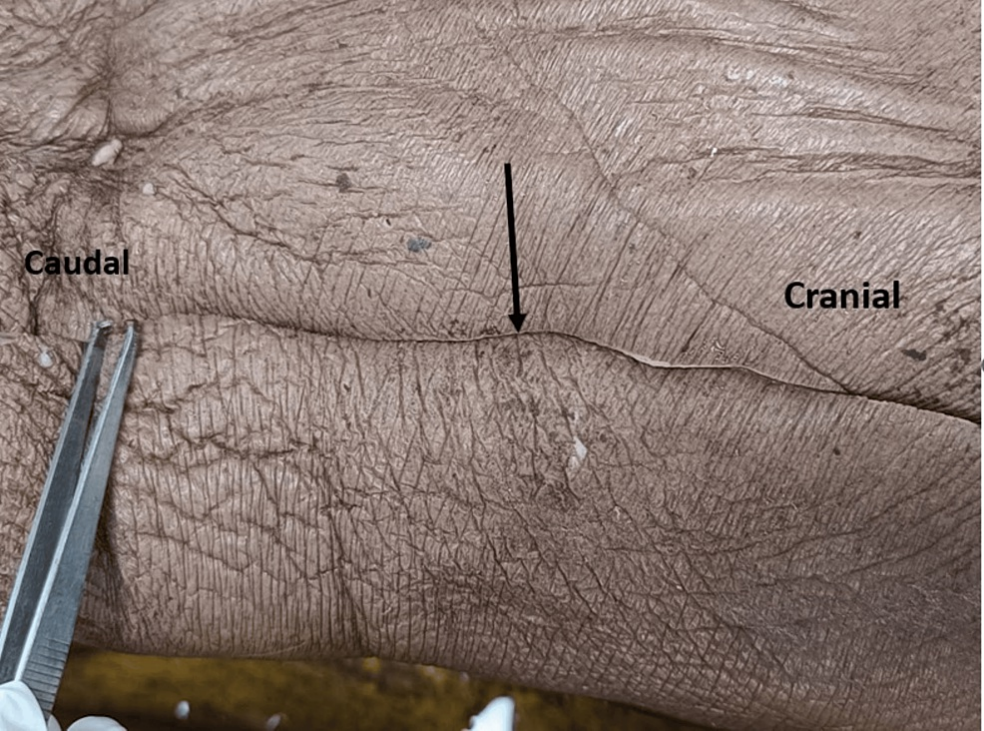
The cadaver is placed in a lateral decubitus position. Skin incision along the midaxillary line (black arrow).

The anatomical knowledge gained through cadaveric dissection and ongoing surgical experience was utilized to determine the finer technical details of this surgery. For the cadaveric anatomical details, we placed the cadaver in a lateral decubitus anatomical position. Skin marking was done along the midaxillary line, which is the vertical line running from the armpit down the side of the body, to highlight the concerned area (Figure 5).

**Figure 6:**
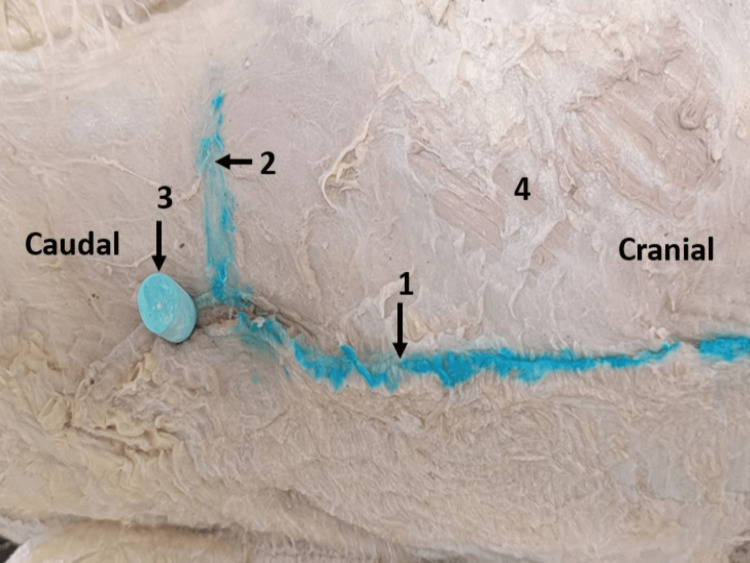
Dissection of the cadaver in lateral decubitus position at the point of the newly proposed primary port. 1. Midaxillary line. 2. Transverse line across the lower border of the 12th rib. 3. One cm below the point of intersection of the lines 1 & 2. At point number 3, we made the transverse incision anteromedially. 4. External oblique muscle.

A transverse blue line was drawn passing across the lower border of the costal margin and its intersection with the midaxillary line identified (Figure 6).

**Figure 7:**
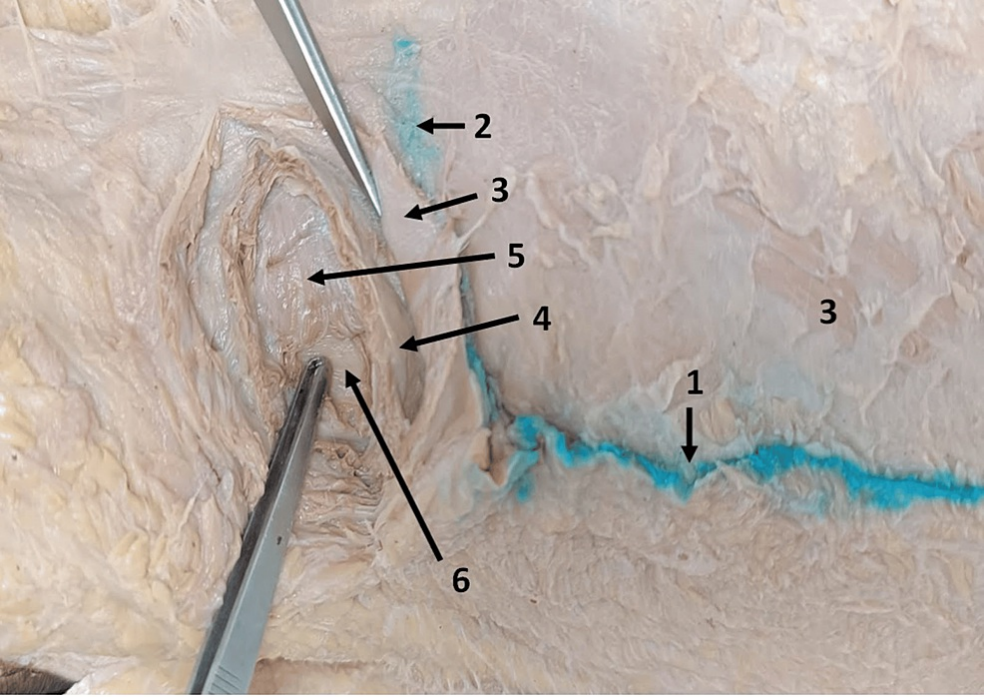
Progressive dissection of the cadaver at the newly proposed site of the primary port. 1. Midaxillary line. 2. Transverse line across the lower border of the 12th rib. 3. External oblique muscle. 4. Internal oblique muscle. 5. Transversus abdominis muscle. 6. Fascia transversalis.

A blue marker was placed just 1 cm below this meeting point on the midaxillary line; this point signifies the site of the primary or camera port during the operative procedure. From this point, a transverse incision of 4-5 cm was made anteromedially on the cadaver. After separating the skin and subcutaneous tissue, upon further layer-by-layer meticulous dissection from superficial to deep, we identified the following layers in sequence: external oblique muscle (following downward, forward, and medial directions); internal oblique (oblique and upward direction of the muscle fibers); transversus abdominis (follow a transverse direction); fascia transversalis; and peritoneum (Figure 7).

**Figure 8:**
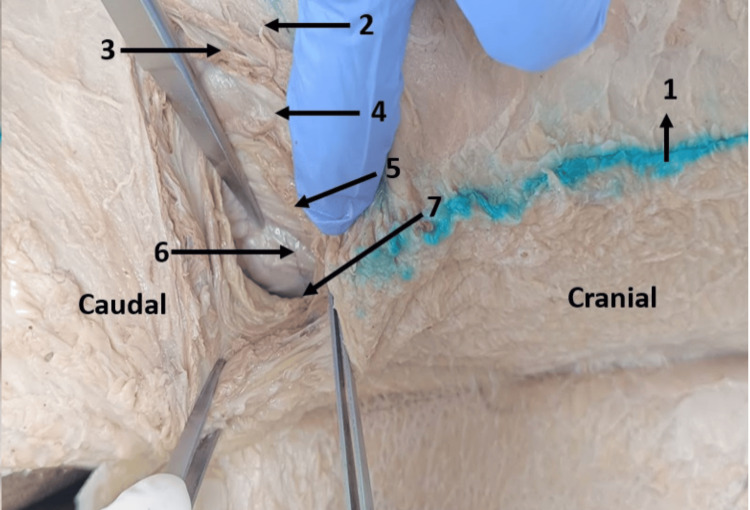
Completed dissection of the cadaver at the newly proposed site of the primary port till the retroperitoneal space. 1. Midaxillary line. 2. External oblique muscle. 3. Internal oblique muscle. 4. Transversus abdominis muscle. 5. Fascia transversalis. 6. Peritoneum. 7. Retroperitoneal space.

After pushing the peritoneum anteriorly, we accessed the retroperitoneal space where we first encountered the paranephric fat and the adipose tissue surrounding the kidney, followed by the renal fascia (Figure 8).

We dissected a total of 15 cadavers for this study. The male-to-female ratio was 4:1. The mean age of the cadavers was 55 years. Comprehensive cadaveric anatomical observation and hands-on experience in the concerned area of operative procedure empower clinicians to perform surgeries superbly, leading to excellent postoperative outcomes.

## Discussion

In our study, we inferred that nephrectomy was comparatively easier in cases such as reflux nephropathy, moderately difficult in cases with pelviureteric junction obstruction due to limited space, more difficult when etiology was ureteric stone, and extremely difficult in cases with large renal pelvic stone due to chronic pyelonephritis or pyonephrosis. There were more complications such as vascular injury, greater blood loss, and increased conversion rate to open surgery when the etiopathogenesis was renal calculus disease with chronic pyelonephritis and pyonephrosis. Therefore, the operating surgeon during the initial phase of retroperitoneoscopy should wisely choose the cases according to their etiopathogenesis. These are primarily our findings and are not backed by statistical significance due to the low number of patients.

In our study, the complication encountered was a peritoneal breach in a case of large ureteric calculi, which was densely stuck to the peritoneum but it was overcome successfully with the application of a 10 mm hem-o-lock clip. In another case, where the etiology was large renal pelvic calculi with chronic pyelonephritis, we had inadvertent renal vein injury, which was also managed successfully by applying traction on the kidney and clipping the vein just below the perforation. Rassweiler et al. also had a similar experience in their initial series of retroperitoneoscopy cases where they encountered significant complications such as bleeding hematoma, pulmonary embolism, or pancreatic fistula in 14% of cases, and a conversion rate of 10%. Their sample size was large, including 200 cases, while in our study, we had significant complications in 12.5% of cases and a conversion rate of 25%, as our sample size was much smaller [6].

Ono et al., in their series of 20 cases, encountered bleeding from an injured capsular artery as the only complication. The mean operative time in their study was around 198 minutes and the estimated blood loss was 135 ml, while in our study, it was 180 minutes and 168 ml on average [7].

Modi et al., in the initial phase of learning, reported a higher conversion rate, longer operative time, and more complications. In their initial 20 cases, the success rate was 60%, blood loss was around 130-180 ml, the duration of surgery was about 100-225 minutes, and there was a failure to progression in 30% of cases [8]. Gill et al. mentioned that 71% of the complications occurred in the first 20 patients [9]. Rassweiler et al. observed that operative duration was 188 minutes, 6% of patients had complications (bleeding in 4.6%, bowel injury in 0.6%, hypercarbia in 0.4%, and pleural injury and pulmonary embolism in 0.2%), and 46% of patients required conversion to open nephrectomy [10]. Hemal et al. reported complications in a group of 185 patients undergoing retroperitoneoscopic nephrectomy and nephroureterectomy for benign conditions. Eighteen patients (10%) required conversion to open surgery due to either vascular complications or failure to progress [11]. Vascular complications were about 2%, with the overall complication rate being around 20%, frequently seen during partial nephrectomy [12,13]. In our study, as our emphasis was mainly to highlight the technical intricacies and the sample size was small, we had major complications in 12.5% of patients, which was less or similar to other groups, and the conversion rate was also less (25%) than other groups. The mean operative duration (180 minutes) and average blood loss (168 ml) were also comparable to these studies.

Kunwar et al., in their initial exposure to retroperitoneoscopic surgery in benign renal diseases, observed that even for extended surgeries, the retroperitoneoscopic approach was safer than the transperitoneal approach with fewer anesthesia-related complications [14].

El-Tohamy et al. also concluded that during retroperitoneoscopic surgeries, cerebral functions, pulmonary functions, and hemodynamic parameters are better preserved than the transperitoneal approach [15]. We also had similar observations in cases with large renal calculi associated with pyonephrosis where in spite of prolonged operative duration (five hours), there were no anesthesia-related complications. Bauza et al. studied the role of the retroperitoneoscopic approach for urolithiasis in 22 patients and inferred that it could be used in combination with other endourological techniques and there is no risk of any peritoneal contamination and reduced incidence of postoperative gastrointestinal symptoms similar to our observation as none of the patients in our study had paralytic ileus or any other bowel-related complications [16].

Tepeler et al., in their observation during the study of 108 patients with benign renal conditions, mentioned that despite being technically challenging and longer duration of surgery in initial phases, retroperitoneoscopic nephrectomy can be performed safely in expert hands. The operative duration in their study (123 ± 38 minutes) was comparable to our study, though, in our study, the conversion rate was higher (25% vs. 11%) mainly because of the small sample size. Intraoperative complications encountered by them were also repaired laparoscopically, similar to our study, without any need for conversion to open surgery [17].

Challacombe et al., in their study of 11 patients with nonfunctioning kidneys with giant hydronephrosis, mentioned the technical challenges such as very little working space and dense perirenal adhesions. Similar difficulties were encountered by us in a case of ureteric calculi with gross hydronephrosis where dissection was very difficult. They suggested technical modifications to overcome these challenges, such as dissection with a balloon in two directions and primary intact dissection, followed by puncture and aspiration of contents. These modifications were very helpful in the successful completion of our surgery [18].

Points in favor of retroperitoneoscopic laparoscopic nephrectomy are as follows [19]: the kidney is a retroperitoneal organ closer to the posterolateral abdominal wall than the anterior abdominal wall. Patient positioning and anatomical structures encountered are similar to the open surgical approach to the kidney. This procedure gives direct and early access to vascular structures and the ureter, so in case of any contingency, we can immediately control the arterial supply to the organ. In transperitoneal laparoscopic nephrectomy, the structures encountered at the hilum from anterior to posterior are the vein, artery, and pelvis. Thus, to visualize the artery, the vein must first be completely mobilized. Many times while mobilizing and dissecting the vein, the lumbar vein gets detached or injured, leading to massive bleeding that is difficult to control. Conversely, in the retroperitoneoscopic approach, the first vascular structure encountered is the renal artery. In contrast to transperitoneal laparoscopic surgery, wherein on the right side the liver and duodenum have to be mobilized along with the colon, and on the left side the tail of the pancreas and the colon, these structures can all be avoided in the retroperitoneoscopic approach. Because retroperitoneoscopic surgery is performed in space posterior to the peritoneum, there is no spillage of blood, so there is no risk of peritonitis as is often seen in transperitoneal surgery. As such, there is less postoperative ileus, facilitating quicker recovery. Because the retroperitoneum is a closed space posterior to all intraperitoneal structures, any minor bleeding or postoperative collection remains confined and stops due to pressure. In transperitoneal laparoscopic surgery, the pneumoperitoneum, due to CO2 insufflation, exerts its pressure effect on all intraperitoneal structures. The retroperitoneoscopic approach is devoid of any such risk. It is of greater benefit in patients with intra-abdominal adhesions, ventral hernias, colostomies, and those on peritoneal dialysis. Unlike the transperitoneal approach, any previous history of open surgery and peritonitis is not a contraindication to this approach.

Challenges encountered in retroperitoneoscopic surgery are as follows: the retroperitoneal space is considerably more limited (≈1 liter) than in the transperitoneal approach (≈3-4 liters); therefore, transperitoneal surgery provides a three-fold larger working space, making retroperitoneoscopic procedure a challenging surgery. Due to the limited space, technically challenging port positioning, entirely different anatomy, and fewer landmarks, there is a steeper learning curve for retroperitoneoscopic nephrectomy. Berglund et al., in a comparative study between the retroperitoneoscopic and transperitoneal approach to nephrectomy in a cohort of extremely obese patients, had inferred that the retroperitoneoscopic group had lesser blood loss, lesser conversion rate, lesser hospital stay, and shorter duration of surgery. In our study also, in one of the extremely obese patients with renal pelvic calculi, we successfully completed the procedure in three hours only and the patient had an uneventful postoperative recovery [20]. Garg et al., in their review article, concluded that with quicker renal access and hilar control, lesser blood loss, early resumption of bowel movements, and fewer gastrointestinal complications, it is advantageous as compared to transperitoneal nephrectomy but the technical difficulty and learning curve still pose a challenge to its popularity [21].

Troubleshooting the challenges

Most challenging are the cases of pyonephrosis with percutaneous nephrostomy tube wherein adhesions of both perinephric and paranephric fat to adjacent organs make it exceedingly difficult to access the renal hilum [19]. The nephrostomy tube has to be removed initially, and there are fibrous nephrostomy tract tissue bands that fix the kidney to the posterolateral abdominal wall, which must be digitally broken before initiating the procedure, otherwise, dissection will be complex and may ultimately lead to conversion. Perirenal adhesions should be divided with coagulating current, and in cases with thick densely adherent fat and diffuse bleeding, procedures like radical nephrectomy have to be performed, and many times, subcapsular nephrectomy may be needed. Another challenge is gas leakage from the primary port, which can be dealt with by taking a full thickness of suture including muscle sheath, by tying the elastic rim of surgical gloves around the port so that it is nicely fixed and does not move, or by placing the gauze piece around the port and tightening the sutures again. To address peritoneal perforation, which is if there is any rent in the peritoneum during dissection, if it is small in size, it can be managed by applying two clips on the peritoneum or by putting a Veress needle in the peritoneal cavity to vent out CO2 at the cost of loss of gases but the benefit of maintaining the space. If it is a large rent, then the opening should be enlarged to make a single cavity, followed by proceeding as per the transperitoneal procedure. In cases where dense adhesions are present, then hilar control is gained first and the ureter is also clipped and divided, a modified Gibson incision is made, a gloved hand is inserted in the retroperitoneum, and a hand-assisted procedure is continued under vision, and then the specimen is retrieved through the same incision. For the prevention of subcutaneous emphysema, the primary port incision should not be bigger than 1.5 times the diameter of the port (π x diameter/2), and the port fixation suture should be tightened to the external oblique muscle sheath.

## Conclusions

The purpose of this study was to elucidate the finer technical and anatomical details used in retroperitoneoscopic nephrectomy surgery. There is no clear-cut recommendation available in the literature regarding the site and method of primary camera port placement, which is the pivotal step, or the finer details regarding surgical landmarks, leading to lesser acceptance of this technique as compared to transperitoneal laparoscopic nephrectomy. Thus, after elaborate and extensive knowledge gained through cadaveric dissection and surgical experience, we hope to impart sound knowledge about various anatomical planes encountered and handling of complications if and when faced. This study is an attempt to throw light on the approach and challenges experienced at our center while performing this procedure in an initial few cases with the hope of making urologists more amenable to the retroperitoneal space.
